# *In Silico/In Vivo* Insights into the Functional and Evolutionary Pathway of *Pseudomonas aeruginosa* Oleate-Diol Synthase. Discovery of a New Bacterial Di-Heme Cytochrome C Peroxidase Subfamily

**DOI:** 10.1371/journal.pone.0131462

**Published:** 2015-07-08

**Authors:** Mónica Estupiñán, Daniel Álvarez-García, Xavier Barril, Pilar Diaz, Angeles Manresa

**Affiliations:** 1 Unitat de Microbiologia i Parasitologia Sanitàries, Facultat de Farmàcia, University of Barcelona, Barcelona, Spain; 2 Department of Microbiology, Faculty of Biology, University of Barcelona, Barcelona, Spain; 3 Departament de Fisicoquímica, Facultat de Farmàcia, Universitat de Barcelona, Barcelona, Spain; 4 Institut de Biomedicina de la Universitat de Barcelona (IBUB), PCB-Edifici Hèlix Baldiri Reixac, Barcelona, Spain; 5 Catalan Institution for Research and Advanced Studies (ICREA), Passeig Lluís Companys, Barcelona, Spain; Universidade Nova de Lisboa, PORTUGAL

## Abstract

As previously reported, *P*. *aeruginosa* genes *PA2077* and *PA2078* code for 10S-DOX (10S-Dioxygenase) and 7,10-DS (7,10-Diol Synthase) enzymes involved in long-chain fatty acid oxygenation through the recently described oleate-diol synthase pathway. Analysis of the amino acid sequence of both enzymes revealed the presence of two heme-binding motifs (CXXCH) on each protein. Phylogenetic analysis showed the relation of both proteins to bacterial di-heme cytochrome c peroxidases (Ccps), similar to *Xanthomonas sp*. 35Y rubber oxidase RoxA. Structural homology modelling of PA2077 and PA2078 was achieved using RoxA (pdb *4b2n*) as a template. From the 3D model obtained, presence of significant amino acid variations in the predicted heme-environment was found. Moreover, the presence of palindromic repeats located in enzyme-coding regions, acting as protein evolution elements, is reported here for the first time in *P*. *aeruginosa* genome. These observations and the constructed phylogenetic tree of the two proteins, allow the proposal of an evolutionary pathway for *P*. *aeruginosa* oleate-diol synthase operon. Taking together the *in silico* and *in vivo* results obtained we conclude that enzymes PA2077 and PA2078 are the first described members of a new subfamily of bacterial peroxidases, designated as Fatty acid-di-heme Cytochrome *c* peroxidases (FadCcp).

## Introduction

*P*. *aeruginosa* is a gram-negative rod known for its metabolic versatility, which allows its distribution in numerous environmental niches, being host in higher plants, invertebrates and vertebrates [1,2]. *P*. *aeruginosa* can metabolize xenobiotics and also unsaturated long-chain fatty acids (LCFAs), widely spread in the environment, to convert them into hydroxylated derivatives (HFAs) known as oxylipins [3,4].

Hydroxylated fatty acids have a wide range of biological functions, being involved in inflammation, signaling, plant pest defense, germination or fungal reproduction [5–7]. Furthermore, the biotechnological applications of HFAs have extensively been studied, constituting important emulsifying agents in food and cosmetics industries, acting as antibacterial or antifungal substances, or being used as intermediate compounds for fine chemistry industry, with an important role in the pharmaceutical area [4,8,9]. However, the bottleneck of their chemical production is the low reactivity of the fatty acid hydrophobic chain [10]. This problem is solved in nature through several enzymatic strategies including LCFA biotransformation by diol synthases (DS), lipoxygenases (LOX), heme-containing fatty acid dioxygenases (DOX), cyclo-oxygenases (COX), hydratases, allene oxide synthases (AOS) or cytochromes P_450_ (CYP450) [11–15]. Moreover, these oxylipin-releasing enzymes are capable to generate products with high regio- and enantioselectivities from a broad range of substrates [16].

We previously described that a diol synthase activity is responsible for the conversion of oleic acid (OA) into oxylipins in *P*. *aeruginosa* [3,4]. Oleic acid is initially converted into hydroperoxide 10-H(P)OME ((10*S*)-hydroxy-(8E)-octadecenoic acid) by a dioxygenase (DOX), followed by conversion of the hydroperoxide intermediate into 7,10-DiHOME ((7*S*,10*S*)-dihydroxy-(8E)-octadecenoic acid) by an oleate-diol synthase (DS). We recently identified the genes coding for these activities, which are linked in a finely tuned operon. Gene *PA2077* codes for the 10*S*-dioxygenase activity (10*S*-DOX) responsible for the first step of the reaction, whereas *PA2078* encodes the (7*S*, 10*S*)-hydroperoxide diol synthase enzyme (7,10-DS), which allows conversion of 10-H(P)OME into 7,10-DiHOME, in a metabolic pathway unique for *P*. *aeruginosa* [3].

Here we report the results of a detailed *in silico/in vivo* study of the nucleotide and amino acid sequences of PA2077 and PA2078, including comprehensive insights into their functional and structural features, with rational mutagenesis analysis of important residues, identification of probable evolutionary elements and phylogenetic analysis. From the results obtained we conclude that proteins PA2077 and PA2078 would be the first described and functionally characterized members of a new di-heme cytochrome c peroxidase enzyme subfamily, acting on long-chain fatty acids.

## Materials and Methods

### Materials

Oleic acid 99% (Sigma) was used as a substrate for bioconversion assays in LC/MS analysis. (10*S*)-hydroperoxy-8E-octadecenoic acid (10*S*-H(P)OME), provided by Dr. Martín-Arjol, was purified from *P*. *aeruginosa* 42A2 culture supernatants as described previously [8,17].

### Bacterial strains

Bacterial strains and plasmids used in this work are listed in S1 Table. All strains were routinely grown in TSB (17 g casein peptone, 3 g soymeal peptone, 2.5 g glucose, 5 g NaCl, and 2.5 g KH_2_PO_4_) at 37°C on a rotary shaker operated at 200 rpm. Antibiotics were added for *P*. *aeruginosa* mutant recombinant strains growth when required at the following concentrations: tetracycline 5 μg ml^-1^; chloramphenicol 200 μg ml^-1^, and ampicillin 100 μg ml^-1^ for *E*. *coli* DH5α recombinant strains.

### Site-directed mutagenesis

Site-directed mutagenesis of selected amino acids in the heme binding or putative P_450_ regions of PA2077 and PA2078 (pGEMTe-77 and pGEMTe-78 variants) was carried out using a QuikChange Site-Directed Mutagenesis Kit (Stratagene), with Pfu/Phusion High-Fidelity DNA polymerase (New England BioLabs) and the primers stated in S2 Table. Amplification was performed at 98°C for 30 s followed by 25 cycles (98°C for 10 s, 60°C for 30 s, and 72°C for 3 min) in a thermocycler PTC200, MJ Research or GeneAMP PCR system 2400 (Perkin Elmer). The amplified products were digested with *Dpn*I (Thermo Scientific) for 2 h and checked by agarose gel electrophoresis. *Dpn*I–resistant plasmid molecules were confirmed by sequencing using plasmid internal primers (S2 Table). Sequencing was performed using an ABI PRISM BigDye Terminator v.3.1 Cycle Sequencing kit (Applied Biosystems), available at the Serveis Científics i Tecnològics of the University of Barcelona. Plasmids carrying the desired mutations were transformed into *E*. *coli* DH5α, as described [18].

### Bioconversion assays

500 μl of 10x concentrated crude cell extracts were incubated with 100 μM oleic acid (OA) or 0.1% (v/v) 10*S*-H(P)OME in 0.5 M Tris-HCl buffer pH 7. Reactions were incubated for 2 h at 37ºC with occasional vortexing, and terminated by acidification to pH 2 with 0.5 M HCl. Released products were extracted with ethyl acetate (1:1; v:v), routinely analyzed by TLC (hexane:diethyl ether:acetic acid; 75:15:10), and further identified by LC–MS/MS analysis.

### Protein expression in *P*. *aeruginosa* mutant strains

In order to obtain functional enzymes for purification, *P*. *aeruginosa PA2078* and *PA2077* genes, cloned into pMMB207 fused to the *tac* promoter [3] were over-expressed or co-expressed in mutants ∆PA2078 and ∆PA2077. Cultures were grown to exponential phase (D.O._600nm_ = 0.6), induced with 1 mM IPTG and incubated overnight at 37ºC. Bradford assay [19] was used to measure soluble protein concentration to determine the effect caused by expression of these genes on both mutant strains.

### Computational analysis of ORFs PA2077 and PA2078

The nucleotide sequences of ORFs PA2077 and PA2078 and their respective orthologous genes were retrieved from *Pseudomonas aeruginosa* database (www.pseudomonas.com) [20]. The recently published nucleotide sequences of *P*. *aeruginosa* 42A2 oleate-diol synthase operon (GenBank #KJ372239) were also included in the analysis [3]. Nucleotide and amino acid sequences were submitted to the basic local alignment search tools BLASTn and BLASTp respectively, to identify possible homologues in the databases available at EMBL/EBI and NCBI (http://www.ncbi.nlm.nih.gov/), and to retrieve identity and similarity percentages by pairwise alignment [21].

Elements involved in enzymatic processing like post-translational modifications were predicted using PSORTb v. 2.0 for bacterial sequences (http://www.psort.org/) [22]. SignalP 4.1 server (http://www.cbs.dtu.dk/services/SignalP) was used for prediction of protein subcellular location [23]. Transmembrane helices prediction was achieved using TMPred Server (http://www.ch.embnet.org/software/TMPRED_form.html) [24]. ExPASy proteomics server (http://us.expasy.org/tools/protparam.html) was used to analyze the protein physico-chemical parameters (ProtParam tool) and to predict isoelectric point and molecular mass of non-processed and mature proteins. GPMAW tool was used for detection of aromatic amino acids [25]. Nucleotide and amino acid sequence alignments were obtained using T-Coffee (http://tcoffee.crg.cat/) [26] or ClustalO (http://www.ebi.ac.uk/Tools/msa/clustalo/) [27] multiple sequence alignment software.

### Search for functional similarity motifs

InterProScan 5.2 was used for domain identification [28]. HMMER (http://hmmer.janelia.org/) was used to create a Hidden Markov Model in order to visualize conserved protein motifs [29]. Amino acid sequences of both proteins were compared with protein sequences of functionally characterized enzymes bearing similar catalytic mechanisms: diol synthases, allene oxide synthases, cytochrome P450 monooxygenases, lipoxygenases, cyclooxygenases, heme-dioxygenases, *cis-trans* isomerases or catalase-peroxidases, available in the literature and in UniprotKB or Protein Data Bank (PDB) (http://www.rcsb.org/pdb/), always considering their catalytic residues [30,31].

Selected protein sequences were used to analyze the phylogenetic relationship between functionally related proteins using MEGA 6 software [32]. A phylogenetic study by maximum likelihood, employing WAG+G as the amino acid substitution model from MEGA 6 software [33] was performed to analyze specific relationships of proteins PA2077 and PA2078 with previously characterized di-heme Ccp enzymes.

### 3D homology model construction and optimization

PA2077 and PA2078 amino acid sequences were compared by BlastP PSI-BLAST (Position-Specific Iterated) using a BLOSUM 45 matrix with PDB database [21] in order to identify homologous proteins with available 3D structure. Template for 3D model construction was selected as that showing the highest identity score (RoxA, pdb *4b2n*) compared with target proteins in multiple amino acid sequence alignments obtained by T-Coffee [26]. For alignment correction, secondary structure prediction was performed using the PSIPRED protein structure prediction server (http://bioinf.cs.ucl.ac.uk/psipred/) [34]. Alignment of conserved amino acid motifs and modeling corrections of the protein core were performed by comparison with the structure of the selected template sequence, using the visualization tool Pymol Molecular Graphics System, Version 1.5.0.4, Schrödinger, LLC (http://www.pymol.org).

A 3D homology model based on sequence alignment between proteins PA2077 and PA2078 and the template RoxA was obtained using Modeller 9.10 [35]. Both heme groups were included in the model generation but no other special restrictions were applied. The coordinate PDB files were used for structure comparison and overlapping structures were monitored using Pymol Molecular Graphics System, Version 1.5.0.4, Schrödinger, LLC.

### Significant evolutionary elements

Presence of palindromic elements in both ORFs was confirmed with Emboss 6.2.0. (http://emboss.bioinformatics.nl/cgi-bin/emboss/palindrome) [36]. Further phylogenetic analysis were performed to establish a relationship pattern between the palindromic sequences found in *PA2077* and *PA2078* and orthologous nucleotide sequences, employing the JC model from MEGA 6 software [33].

## Results and Discussion

### Predicted parameters

**Fig 1:**
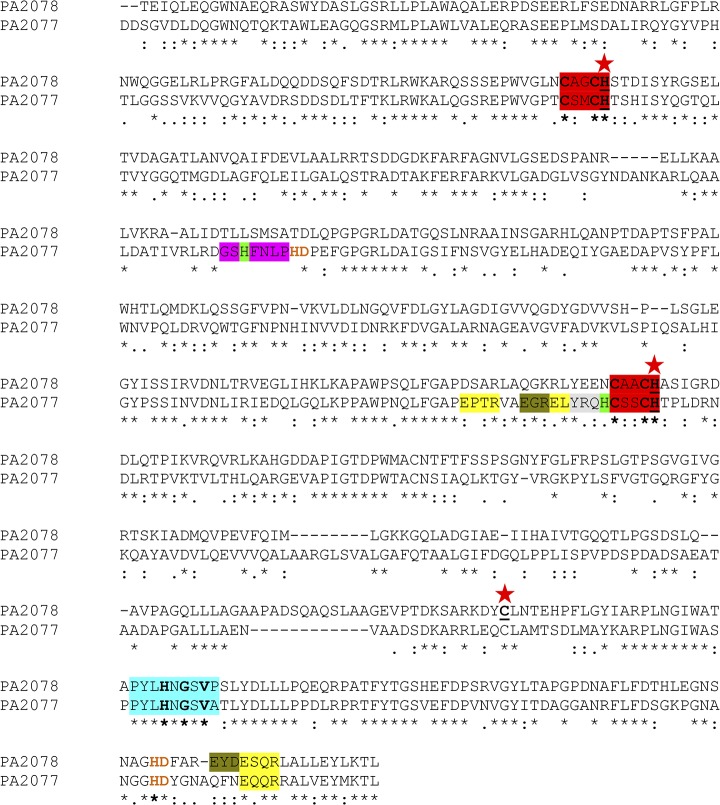
Amino acid sequence alignment of PA2077 and PA2078 proteins, obtained by ClustalO. Significant amino acid motifs are highlighted in squares and the functionally identified amino acids are shown in **bold**. Conserved heme sequences (CXXCH) are shown in red. The predicted motif for ferrous ion union is depicted in green (EGR or EYD). The signature of oxidases containing the essential histidine like in MauG is shown in blue. Predicted tyrosyl radical (YRQH in PA2077) appears in grey. P450 motifs (EXXR) are in yellow and the distal histidines are highlighted in light green. Peroxidase signature (GXHXCLPHD) is shown in pink, with the peroxidase motif (HD) in orange. A red star indicates the position of the mutated residues (underlined).

*P*. *aeruginosa* ORFs PA2077 and PA2078 (UniProt Q9I238 and Q9I237) code for proteins of 634 and 624 amino acids with molecular masses of 68.6 and 67.3 kDa (66.5 and 65 kDa in mature form) and a theoretical *p*I of 5.37 and 5.38, respectively. As inferred from UniProt electronic annotation, the two proteins are described as putative heme-binding proteins with electron transport activity. In agreement with the periplasmic location of oleate-diol synthase activity [37], both proteins display type I signal export sequences that allow transport of the synthesized products to the periplasm, a fact experimentally confirmed by PhoA fusion screening [38]. According to SignalP, signal peptidase cleavage site is located at positions 21 and 23 in PA2077 and PA2078, respectively (Table 1). Despite being periplasmic proteins [4,37], presence of a transmembrane helix was predicted by TMpred for PA2077, but not for PA2078, suggesting that while PA2077 could be a periplasmic membrane-related protein, PA2078 would stay completely soluble in the periplasm. As observed before, PA2077 and PA2078 display very low homology to other proteins in the databases, except for those corresponding to their orthologues in other *P*. *aeruginosa* strains [3]. Moreover, PA2077 and PA2078 bear 43.4% identity (58% similarity) with respect to each other, probably pointing to a common predecessor during evolution, as previously suggested [3] (Fig 1).

**Table 1:** Features and attributes of PA2077 and PA20778 nucleotide and amino acid sequences.

Attribute	PA2077	PA2078
ORFs (bp)	1905	1875
Molecular mass (KDa)	68.6	67.3
Mature protein	66.5	65.0
Mature protein + 2 hemes	68.7	67.5
P_i_ (theoretical)	5.37	5.38
% aromatic amino acids (nº of F, W and Y amino acids)	8.2 (21/10/20)	7.6 (23/10/15)
Heme attachment^a^ (N terminus/C terminus)	CAGCH_130_/ CAACH_375_	CAGCH_130_/ CAACH_365_
Axial heme ligands^a^ (N terminus/C terminus)	H_130_/H_375_ H_611_	H_130_/H_365_ H_603_
MauG motif	PYFH_553_NGSVP	PYFH_543_NGSVP
F_317_ equivalent	F_269_	F_260_
W_302_ equivalent	W_251_	S_243_
Signal Peptide cleavage site	21	23
IR in protein-coding sequence (+ strand)	GACGTCGGCG	CCATCTGCAA
IR length	57 pb	68 bp

^a^: Numbering includes signal peptide.

### Functionally significant sequence motifs in PA2077 and PA2078

Rational comparison of amino acid sequences among previously characterized oxylipin-forming enzymes allowed identification in PA2077 and PA2078 of several significant motifs or specific residues (Fig 1) related to heme/iron-binding sites or relevant in oxygenation reactions. Thus, a sequence (**Y**R*Q* **H**) similar to the tyrosil radical (**Y**R**WH**) involved in dioxygenation of fungal linoleate-diol synthase activity [39] could be found in PA2077 but not in PA2078. The latter displays several consensus motifs like an **EYD** sequence presumably related to iron binding, the **HD** motif found in peroxidases, and a possible P450 sequence (**E**SQ**R**), all of them located in the 22 C-terminal amino acid positions of the protein (Fig 1). PA2077 contains additional conserved amino acid motifs like three putative P450 sequences (**E**PT**R, E**LY**R** and **E**QQ**R**), two **HD** peroxidase motifs, and a signature very close to the **G**S**H**F*N* **L**P**HD** peroxidase consensus sequence [40]. However, the sequence FXX**G**P**H**X**CL**G, responsible for hydroperoxide isomerase activity in *Aspergillus fumigatus* linoleate-diol synthase [41] could neither be found in PA2077 nor in PA2078. Interestingly, both proteins bear a leucine as the C-terminal residue (Fig 1), a hydrophobic amino acid close to the isoleucine shown to play an essential role in *P*. *aeruginosa* lipoxygenase (LOX) activity [42].

Search for conserved functional amino acid domains in discontinuous pattern mode allowed further identification of short, relevant elements shared by both proteins (Fig 1), such as the presence of two conserved cytochrome c-like domains of 19 amino acids (368–386) in PA2077 and 21 amino acids (349–370) in PA2078 that exhibit identity to cytochrome c oxydases (cbb3-type) [43]. Also, the two heme-binding motifs (**C**XX**CH**), used for covalent attachment of heme in bacterial di-heme cytochrome c peroxidases (Ccps) were found in both proteins. Moreover, the signature ‘PYL**H**N**G**S**V**’, containing the essential histidine of oxydases and described for MauG (methylamine dehydrogenase) as a cytochrome c peroxidase domain [44], was also found in PA2077 and PA2078 (Fig 1, Table 1). These findings suggest that the two proteins involved in *P*. *aeruginosa* oleate diol synthase activity are members of the cytochrome c peroxidase (Ccp) enzyme family.

### Comparison of PA2077 and PA2078 with other bacterial Ccps

**Fig 2:**
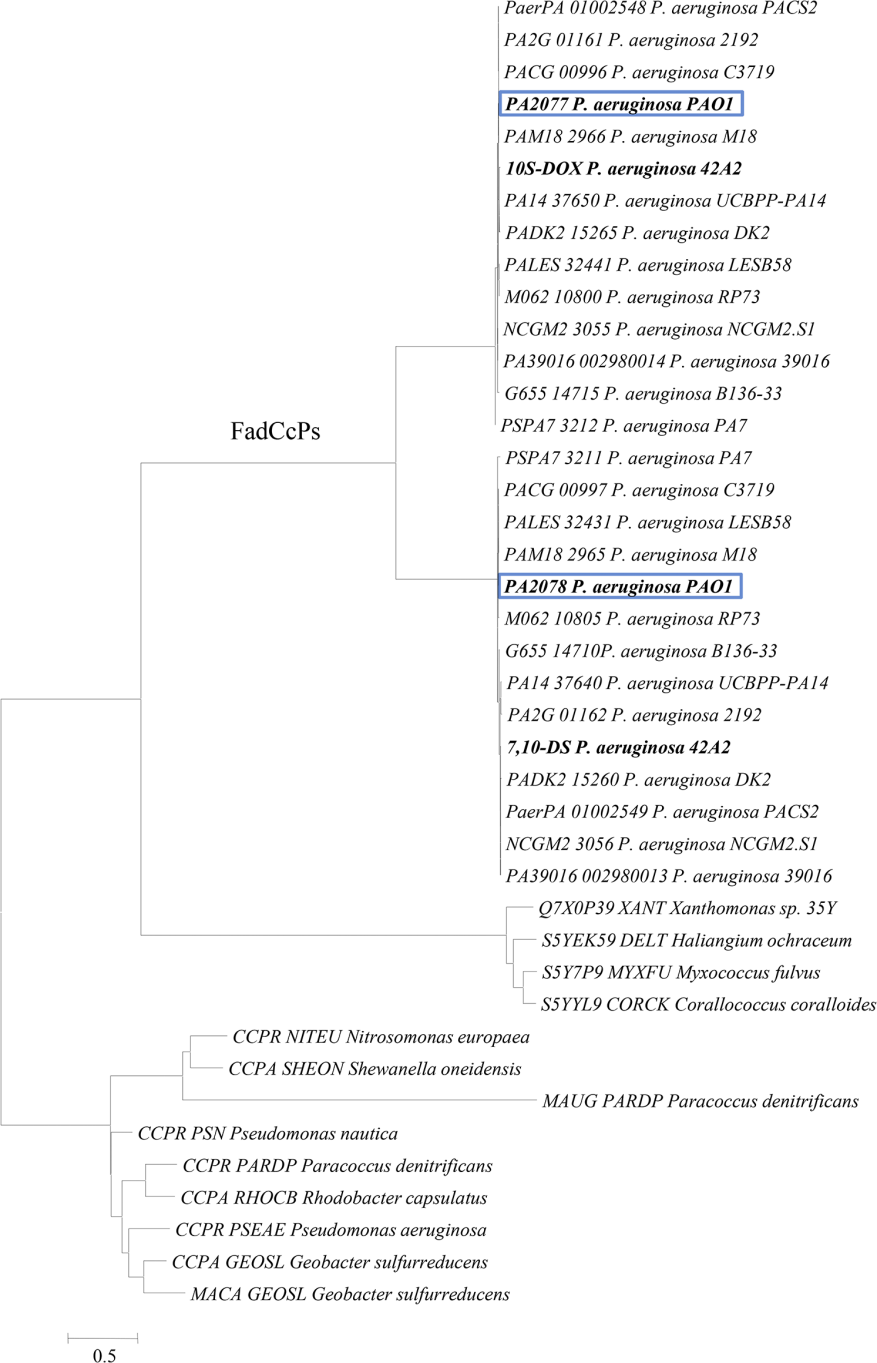
Phylogenetic relationship between amino acid sequences of bacterial di-heme cytochrome *c* peroxidases (Ccps). NITEU–*Nitrosomonas europaea*, SHEON–*Shewanella oneidensis*, PSN–*Pseudomonas nautica*, PARDP–*Paracoccus denitrificans*, RHOCB–*Rhodobacter capsulatus*, PSEAE–*Pseudomonas aeruginosa*, GEOSL–*Geobacter sulfurreducens*. XANT–*Xanthomonas sp*. 35Y, DELT–*Haliangium ochraceum*, MYXFU–*Myxococcus fulvus*, CORCK–*Corallococcus coralloides*. Oleate-diol synthase enzymes (PA2077 and PA2078 orthologues) constitute the newly described subfamily of FadCcps (Fatty-acid di-heme cytochrome c peroxidases).

Known bacterial Ccp protein family is predominantly spread among proteobacteria (Fig 2), with an average molecular mass of 35–40 KDa, whose active form purifies as a homodimer [45–48]. However, like rubber oxygenase RoxA from *Xanthomonas* sp. 35Y and its orthologous genes [49], PA2077 and PA2078 show about double molecular size compared to other bacterial Ccps, and display activity as monomers. RoxA was previously classified as a Ccp protein showing homology to PA2078 [49], which allows inclusion of protein PA2078 in the same enzyme family. Moreover, PA2078 and PA2077, share a high degree of identity between them, indicating that PA2077 can also be assigned to the Ccp protein family, even if displaying different biochemical functions [3].

To further prove this preliminary assignment, PA2077 or PA2078 and their orthologs were aligned to obtain two phylogenetic join-trees with either other known oxylipin-forming proteins (not shown) or with the 13 known members of bacterial Ccps (Fig 2). In both cases, PA2077 and PA2078 appeared localized together in the same node of the phylogenetic tree. In the first tree they appeared far related to the other oxylipin-forming enzymes analyzed, which share less than 18% identity with PA2077 and PA2078. In the second phylogenetic tree, two well-differentiated groups of bacterial Ccps appeared (Fig 2). One group includes those proteins functionally characterized as cytochrome c peroxidases, together with MauG (methylamine dehydrogenase), whereas the second protein group includes *Xanthomonas* sp. 35Y RoxA with its orthologs, and *P*. *aeruginosa* PA2077, PA2078 homologous proteins (Fig 2). According to these results, protein PA2077 (10*S*-DOX) constitutes, together with RoxA and MauG, the third functionally characterized bacterial oxygenase-Ccp protein. These evidences further confirm that the proteins responsible for *P*. *aeruginosa* oleate-diol synthase activity are quite unique, not only among bacteria but also with respect to other oxylipin-forming enzymes previously reported like those of *Aspergillus fumigatus* [41,50] or linoleate diol synthase (LDS) from the rice blast fungus *Gaeumannomyces graminis*, which consist on a single protein bearing two domains [51].

### Comparative 3D modeling of PA2077 and PA2078

**Fig 3:**
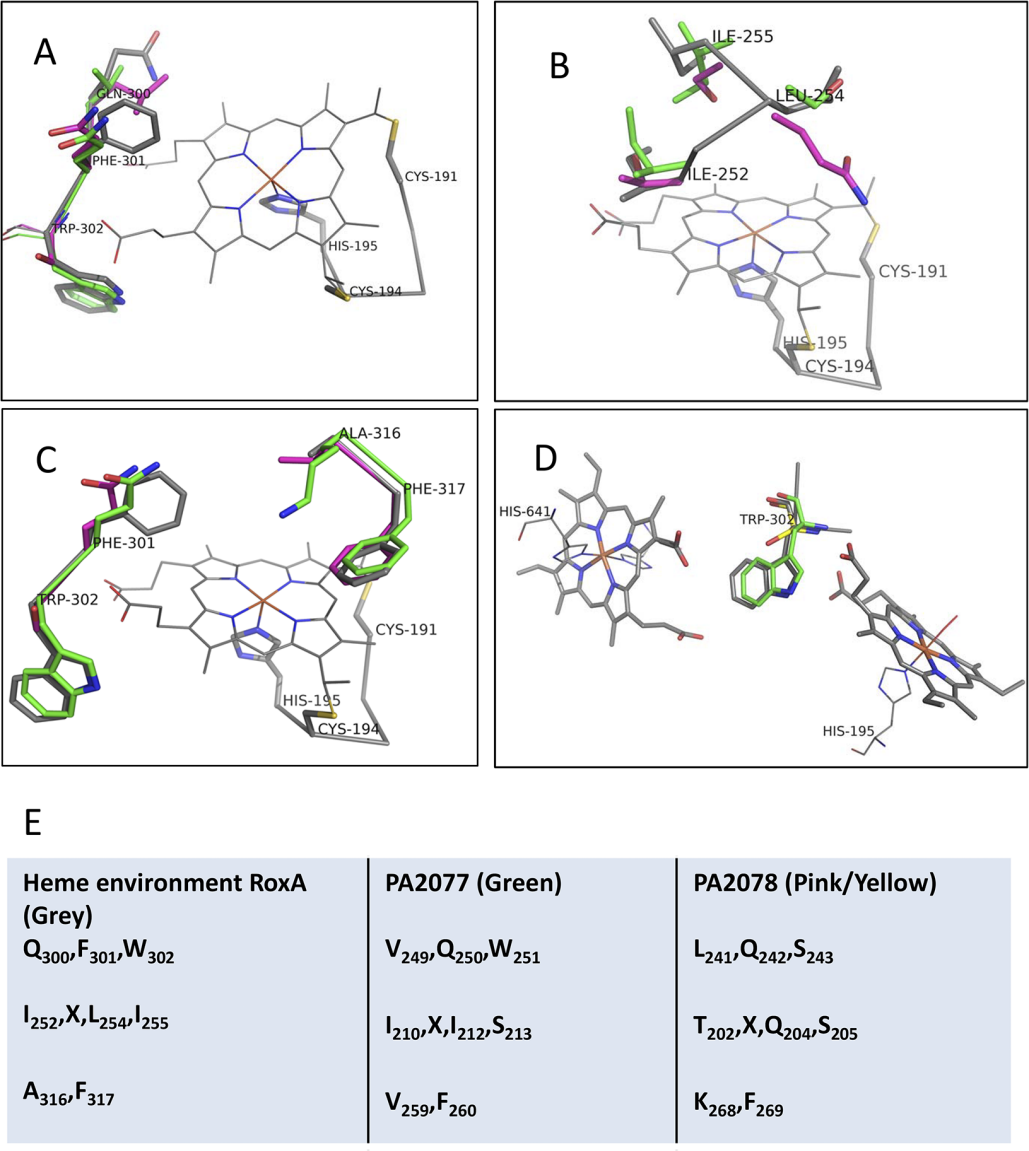
A, B, C, Homology models of surrounding amino acids of the low potential heme environment in PA2077 and PA2078, obtained using rubber oxidase RoxA protein (pdb *4b2n*) as a template (C_191_XXC_194_H_195_). RoxA structure is shown in grey, PA2077 in green and PA2078 in pink. **D,** Amino acid relationships between both heme-binding motifs (H_**195**_, H_**641**_) in RoxA compared to those of PA2077 and PA2078. **E,** Heme environment changes observed in PA2077 (green) and PA2078 (pink/yellow) in comparison with those of RoxA (grey).

Taking into consideration the difficulties found in purifying PA2077 and PA2078 proteins for crystallization purposes, a comparative homology 3D model was obtained here. Based on the results from multiple sequence alignment, pdb *4b2n* from RoxA, the closest crystallographic structure related to PA2077 and PA2078 known so far, was selected as a template for modeling the 3D structure of the two proteins (S1 Fig). RoxA catalyzes the oxidative cleavage of natural rubber (poly-[*cis*-1,4-isoprene]), a complex hydrophobic biopolymer. As other Ccp proteins, RoxA bears di-heme binding domains, which act as low potential (LP; N-terminal domain; -65 mV) and high potential (HP; C-terminal domain; -130 mV to -160 mMV) hemes. Both heme domains are linked by W302, which provides an inter-heme electron bridge. RoxA active site is located in residue H195, acting as a proximal axial ligand to the heme iron. The closely located hydrophobic residues F317, A251, I252, F301, L254, I255, and A316 constitute the heme environment, being F317 essential for activity. Furthermore, the complex flexibility of three hydrophobic amino acid loops located in the distal active site possibly create a transient channel for substrate access and accommodation [52,53]. Although RoxA, PA2077 and PA2078 have a similar molecular mass (71.5, 66.5 and 65 KDa, respectively), they share low percentage of sequence identity (coverage 20% and 32%, identity 43% and 65%, PA2077 and PA2078 respectively in front of RoxA) and, as shown in the multiple sequence alignment (S2 Fig), insertions and deletions occur at different regions of their sequences, suggesting a different spatial loop arrangement. Heme-binding sites are separated by 245 and 235 amino acids in PA2077 and PA2078 respectively, further than the 199 amino acid distance found in RoxA [52], confirming this assumption. The low similarity and complex fold of RoxA, together with ubiquitous loops, made the global process of modeling difficult to validate. Although the surface loops were poorly modelled in the 3D-models obtained, the core region holding both heme groups, well conserved according to the sequence alignment (S2 Fig), provided enough confidence to trust this modelled region for analysis. Therefore, the conserved dual-heme environment core, constituting the common trait of Ccps [52], could be modeled with enough confidence to be studied in detail, providing insights into the catalytic preferences of both proteins. To obtain a reliable model, a multiple sequence alignment including PA2077, PA2078 and RoxA (S2 Fig) was used instead of pairwise alignment, to magnify similarities around the heme cavities. Important amino acid differences could be identified in the oxygen-binding axial heme cavity (His195 in RoxA), which might alter reactivity by modifying the heme active conformation and substrate-binding pocket. As shown in Fig 3, important, non-neutral variations with respect to RoxA were identified in PA2077 and PA2078 heme environments, where hydrophobic residues are mainly substituted by polar amino acids: F301Q in both proteins, L254S in PA2077, and I255S, I252T and L254Q in PA2078. Moreover, a drastic A316K substitution is predicted for PA2077 heme-binding site. Presence of this positive charge, partially stabilized by a serine residue sitting nearby, might be important in the catalytic mechanism of this protein (Fig 3).

**Fig 4:**
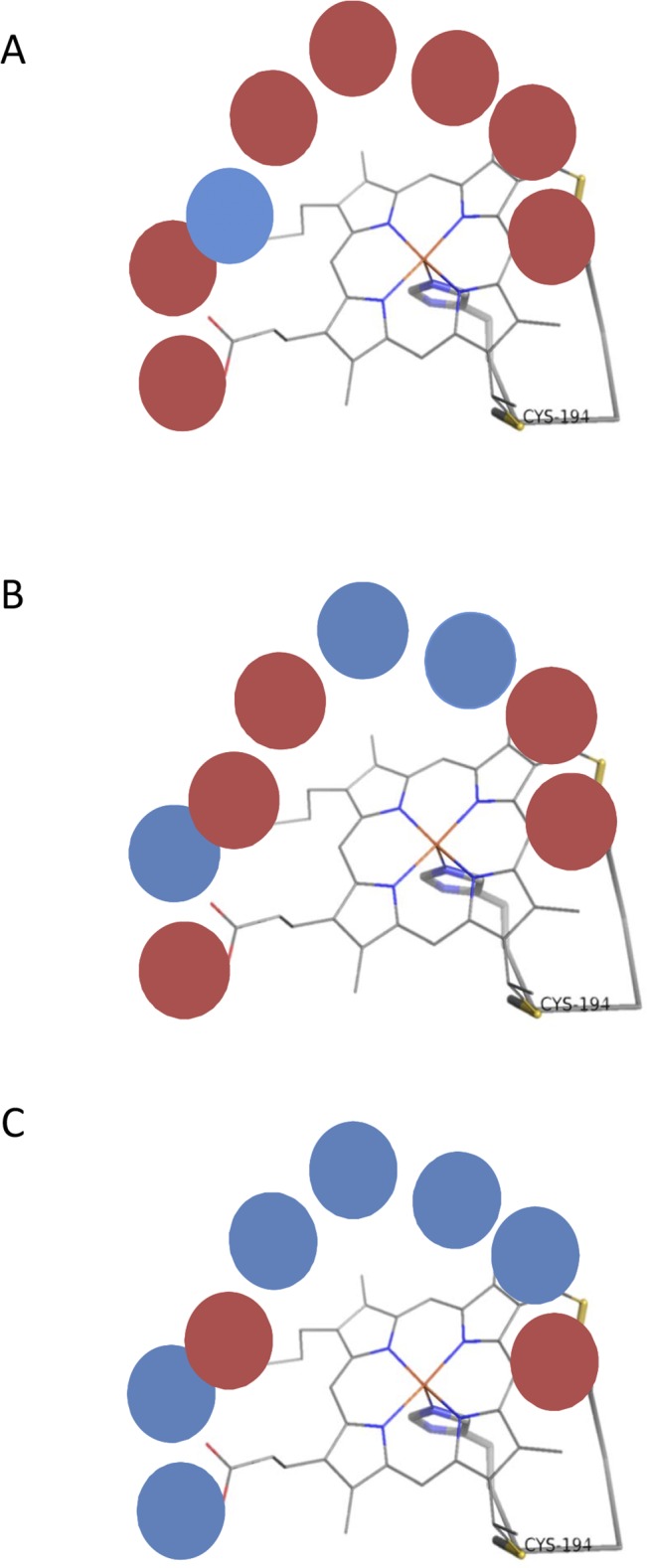
Heme environment hydrophobicity is shown for RoxA (A), PA2077 (B) and PA2078 (C). Polar amino acids are shown as blue circles and hydrophobic amino acids appear in red.

A general heme-binding site scheme for RoxA, PA2077 and PA2078 is depicted in Fig 4. The catalytic N-terminal heme environment of RoxA and PA2077, where the monomeric assembly of the oxygen molecule is produced, is mainly constituted by hydrophobic amino acids (excluding the lysine change in PA2077 previously described). However, a more hydrophilic environment was found in PA2078 model (Fig 4). These features can explain the polar nature of the substrate preferred by each enzyme, as RoxA transforms natural rubber, a highly hydrophobic polymer, and PA2077 substrates are mainly monounsaturated LCFAs with a polar head, whereas PA2078 acts on more polar hydroperoxide fatty acids [3,54]. Supporting the higher substrate specificity similarity of RoxA and PA2077, both proteins conserve a tryptophan located between the two heme groups (W302 in RoxA and W251 in PA2077), which can act as a linker of the redox reaction between the two hemes (Fig 3D; Table 1). On the contrary, presence of a serine instead of a tryptophan at this position in PA2078 (S243) may alter the electron transfer process between the two hemes, which would thus function as a mono-heme enzyme like cytochrome CYP450, AOS or *cis-trans* isomerase (Cti) enzymes [55]. Therefore, the amino acid variations found in the heme-binding structures of the analyzed proteins may explain the catalytic differences in behavior found between these three enzymes.

### Protein evolution elements found in genes *PA2077* and *PA2078*

**Fig 5:**
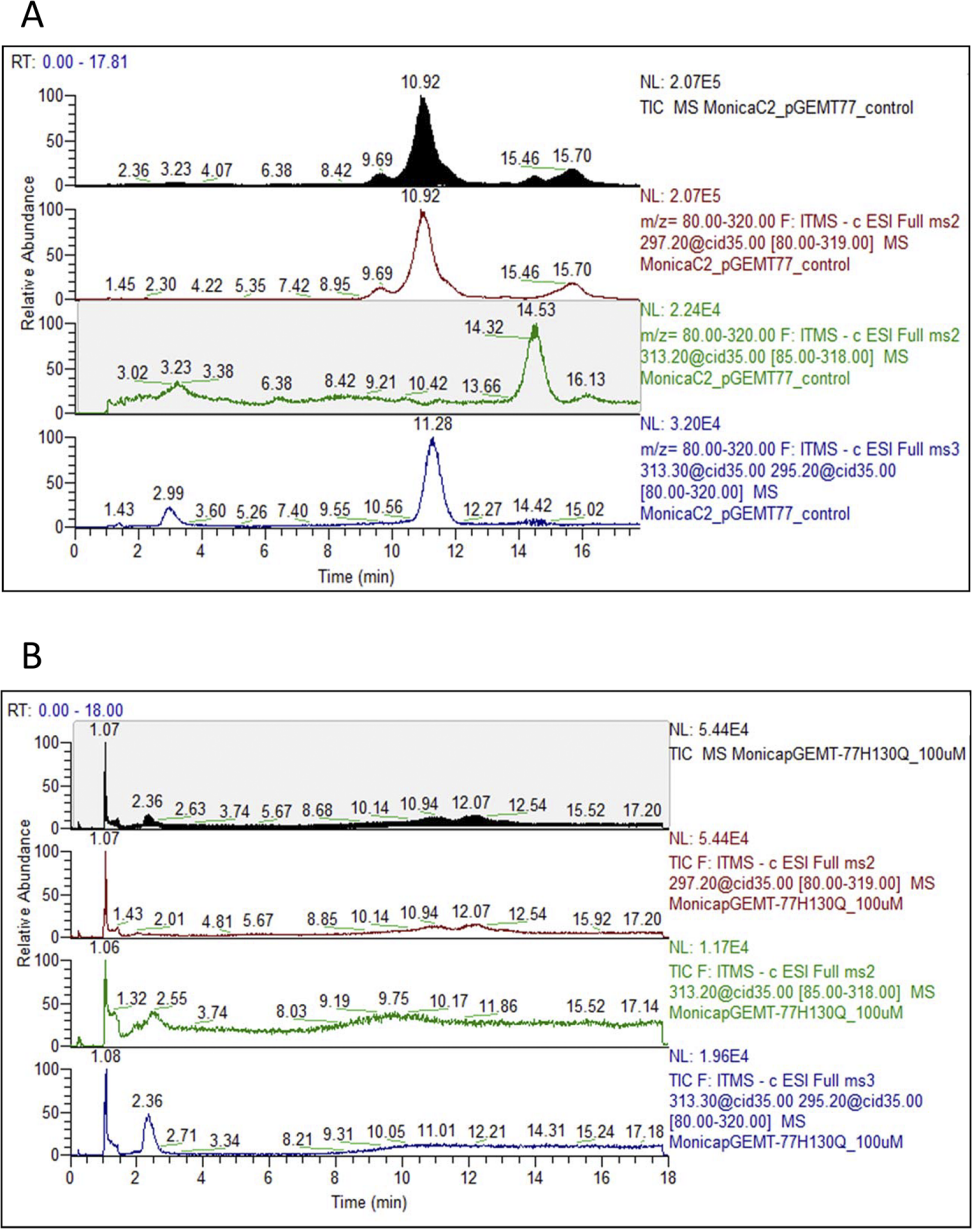
Products released from oleic acid by wild type PA2077 (A) and mutant PA2077 H130Q (B). Hydroperoxide 10-H(P)OME (RT = 11) could only be detected for wild type PA2077, whereas no conversion of oleic acid occurred when both mutants, PA2077 H130Q and PA2077 H375Q, were assayed.

Analysis of the nucleotide sequence of ORFs PA2077 and PA2078 revealed the presence of short inverted repeats (IR) of 19 and 22 amino acids, inserted at positions 716 and 870, respectively (Table 1). On the contrary, no IR insertions were found in *rox*A nucleotide sequence. *In silico* removal of the IR insertions from the original nucleotide sequence of *PA2077* and *PA2078* produced a frame-readout from amino acid 240 in PA2078, which would result in loss of the C-terminal heme-binding motif **C**AA**CH,** supporting the previous idea that PA2078 might not use the distal heme-binding site for activity, acting as a mono-heme enzyme. On the other hand, when the same procedure was applied to *PA2077*, the two **C**XX**CH** motifs were maintained, suggesting that they are required for activity. To prove this hypothesis, mutants of PA2077 and PA2078 at the proximal and distal heme motifs (Fig 1) were experimentally constructed and tested for activity on their corresponding substrates. Mutants at either position H130 or H375 (residue numbering with signal peptide) in protein PA2077 failed to convert oleic acid into 10-H(P)OME (Fig 5), definitely showing that both histidines (the two hemes) are required by PA2077 to display activity. However, mutants H130Q, H365Q and C518S constructed for PA2078 (numbering includes signal peptide) produced the same pattern as wild type PA2078 when assayed for conversion of 10-H(P)OME into 7,10-DiHOME (S3 Fig). These results indicate that neither the proximal or distal heme groups, nor the cysteine putatively related to P_450_ enzymes are involved in the catalytic activity of PA2078. Thus, despite being a di-heme enzyme, none of the heme groups of PA2078 seems to be involved in 10-H(P)OME conversion. Further mutagenesis approaches are required to completely elucidate the catalytic environment of PA2078, which must be located elsewhere in the protein sequence, out from the heme region.

The lack of activity of the heme groups in PA2078 supports the previous hypothesis that these motifs might have been inherited from an ancestor di-heme protein and maintained during evolution independently of the present activity of the enzyme (3). In fact, the in-frame repeat insertions found in protein-coding regions like those of *PA2077* and *PA2078* have been proposed to constitute evolutionary mechanisms for enrichment of protein diversity in other proteobacteria genomes [56,57], and could be responsible for the loss of functionality of the heme groups in PA2078, thus contributing to the high metabolic versatility and adaptability shown by *P*. *aeruginosa* strains.

### Insights into the evolutionary pathway of PA2077 and PA2078

As suggested in a previous work [3] and confirmed here after site directed mutagenesis, genes *PA2077* and *PA2078* would derive from a common phylogenetic ancestor, involving a gene duplication event followed by functional divergence into two different catalytic activities. Moreover, no functionally characterized orthologues exist in the databases or in the literature, thus constituting the first reported elements for oleic acid metabolism in *P*. *aeruginosa* strains [3]. Here we further analyzed the nature of this ancestor and the probable evolutionary model of such a process. From the results obtained above, we can conclude that protein PA2077 conserved the two heme-binding functional domains for dioxygenase activity even after the IR insertions, so it can be assumed that it is more closely related to the ancestral enzyme than PA2078, where the CXXCH motifs can be abolished without losing activity. Moreover, the IR sequence GACGTCGGCG is conserved in most *PA2077* homologous genes, whereas the IR sequence found in *PA2078* varies among the corresponding orthologous genes, which means that they have suffered a higher rate of mutations (Table 1). From both, the experimental and the *in silico* data obtained, we can conclude that the enzymes responsible for *P*. *aeruginosa* oleate diol synthase activity are inparalogs, where the activity of PA2078 would have been more recently acquired than the dioxygenase activity of PA2077, which seems to be much closer than PA2078 to the antecessor protein.

### Evolutionary conservation of diol synthase operon

**Fig 6:**
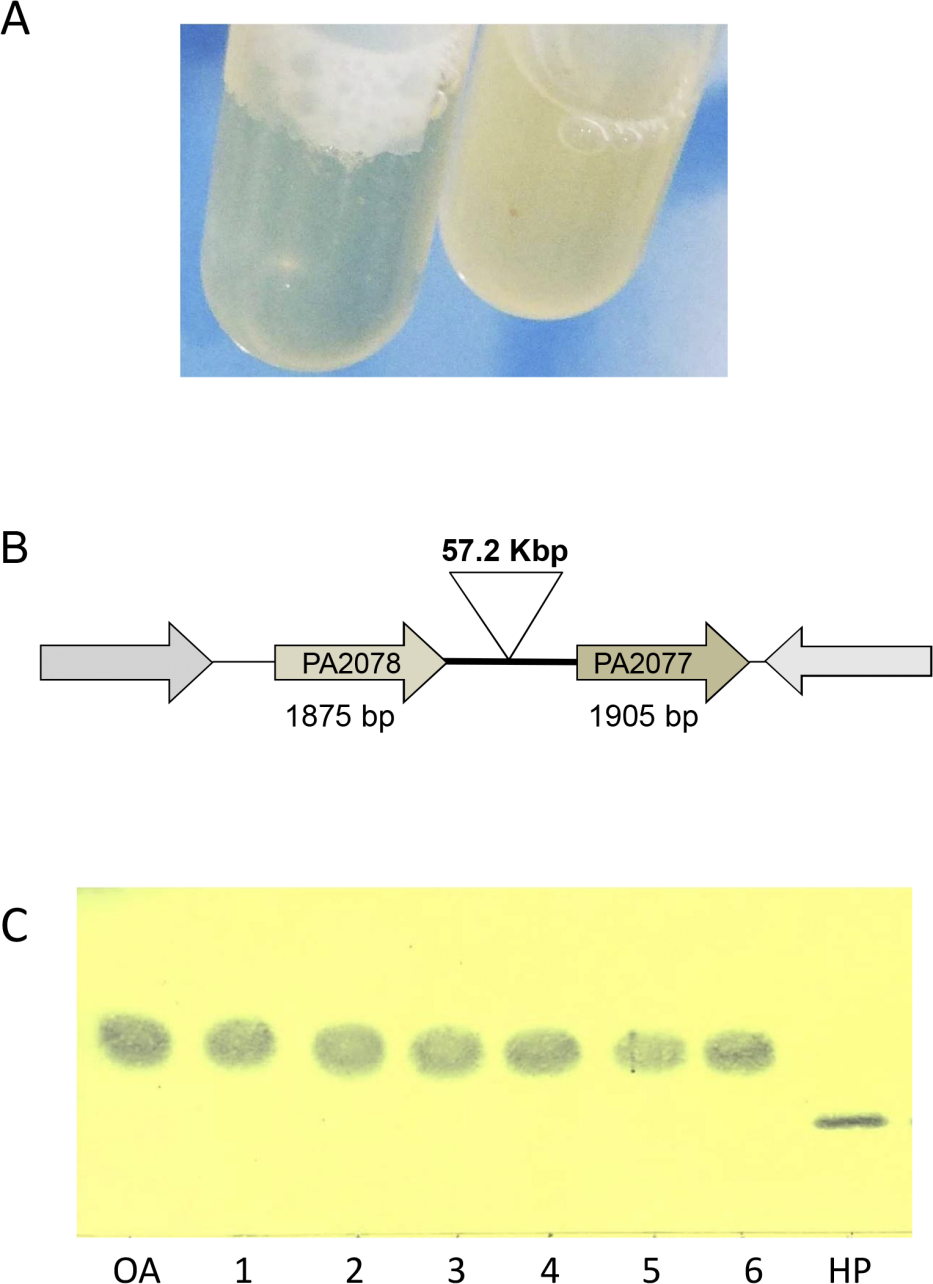
(A) Effect of gene *PA2077* overexpression on cell viability of mutant ∆PA2078, measured as soluble protein and culture cell density. Non IPTG-induced (right tube; 1.21 mg mL^-1^ soluble protein) and IPTG-induced (left tube; 0.8 mg mL^-1^ soluble protein) cultures of mutant ∆PA2078 carrying gene *PA2077*, shown as example of cell lysis caused by overexpression of gene *PA2077*. An overall 66% decrease in soluble protein was found in mutants overexpressing gene *PA2077*, suggesting a toxic effect of the products released by the encoded functional 10(*S*)-DOX. (B) Schematic representation of the *PA2078-PA2077* operon showing the 57 Kbp DNA insertion affecting the operon architecture. (C) Thin layer chromatography analysis showing lack of oleic acid conversion by KK strains (1,2: KK1; 3,4: KK14; 5,6: KK72) incubated for 2 [1,3,5] or 4 hours [2,4,6] with oleic acid. Oleic acid and 10-H(P)OME are shown as control markers.

The evolutionary significance of *P*. *aeruginosa* diol synthase operon was tested by *in vivo* plasmid-based expression of gene *PA2077*, the hydroperoxide-forming enzyme, in the genetic context of mutant ∆PA2078 where the whole operon expression is blocked and there is no conversion of oleic acid at all [3]. However, this construction demonstrated to be lethal and no growth could be obtained (Fig 6A). Moreover, when gene *PA2077* was expressed in mutant ∆PA2077, where protein PA2078 is functional, a similar toxic effect was found, with an overall 66% decrease in soluble protein production (Fig 6A). These results show that overexpression of gene *PA2077* has a toxic effect due to the properties of the hydroperoxide product released by the encoded functional PA2077 (10(*S*)-DOX). On the contrary, overexpression of gene *PA2078* in either mutant under the same conditions caused no decrease of soluble protein concentration compared to the wild type strain. As described before [3], the 10(*S*)-DOX encoded by gene *PA2077* produces oxygenation of OA and could possibly act on other cellular unsaturated fatty acids, leading to accumulation of high concentrations of organic hydroperoxides, which could damage DNA, proteins and membrane phospholipids [42,58] thus causing cell lysis and a concomitant soluble protein reduction. In fact, although conversion of several unsaturated fatty acids has already been explored with the operon PA2078-PA2077 [4], the physiological range of PA2077 substrates, as for other novel dioxygenases, remains still to be elucidated. Taking into consideration these observations, the tandem disposition of genes *PA2078-PA2077* in the diol synthase operon, and its genetic regulation as a dis-coordinated operon (gene *PA2078* is expressed to a double extent than gene *PA2077*), probably allows enzymatic control of organic hydroperoxide production, thus avoiding the cellular damage caused by organic hydroperoxide accumulation. Therefore, the operon nature and disposition of oleate-diol synthase genes (transcribed *PA2078* (2X)→*PA2077*) would have evolved to acquire the differentiated functions of the two encoded enzymes, probably addressed to long chain fatty acid oxygenation (PA2077) and further detoxification/control mechanism (PA2078) to avoid the intracellular effects of hydroperoxide accumulation. This was further confirmed after expression of the complete operon in either a ∆PA2077 or a ∆PA2078 background. Normal growth rates and full activity were achieved in both mutants, indicating that when the two enzymes are co-expressed *in trans*, both maintain their respective functions, with PA2078 abolishing toxicity. These results strengthen the idea that presence of the diol synthase operon represents an evolutionary advantage for bacterial strains, constituting a non-essential metabolic pathway which, nevertheless, can provide benefits to the cell when present [3]. This fact is supported by the recently isolated *P*. *aeruginosa* KK-related strains which contain a non-phagic insertion of 57.2 Kb located upstream *PA2077* (Fig 6B), a phenomenon occurred later after gene duplication, which involves mercuric resistant coding-proteins [59]. These strains constitute the first *P*. *aeruginosa* cells reported so far in which oleate-diol synthase activity was absent when we tested them for bioconversion of oleic acid (Fig 6C). Therefore, interruption of the oleate-diol synthase operon does not affect the viability of the KK-related strains in the environment but prevents them from long-chain fatty acid oxygenation.

### PA2077 and PA2078 constitute a unique, new subfamily of di-heme peroxidases

According to the previous functional, structural and phylogenetic results, we conclude that PA2077 and PA2078 are di-heme proteins that can be classified as bacterial Ccps, analogous to the well-characterized and heterogeneous group of fungal and bacterial diol synthases. They show low homology to other proteins in different databases, preventing construction of a valid complete 3D homology model. However, the 3D model structure of the heme environment allowed identification of relevant differences in residue composition that might justify the substrate specificity of each enzyme. Both proteins have different but complementary enzymatic functions, constituting a set of metabolic elements for fatty acid or fatty acid-derivatives metabolism in the cell periplasm. Nevertheless, the phylogenetic results obtained here indicate that PA2077 and PA2078 do not group in the main phylogenetic branch described so far for functionally characterized Ccps, constituting thus a new cluster of functional and structural enzymes. Therefore, we propose the inclusion of proteins PA2077 and PA2078 as the first functionally characterized members of a new subfamily of enzymes of the Ccp family, for which we suggest the designation of Fatty acid di-heme Cytochrome c peroxidases (FadCcps).

## Supporting Information

S1 Fig Expanded view of the constructed 3D-homology model structures of PA2077 (A) and PA2078 (C). The structure of RoxA (B), used as a template, is shown as a reference. Conserved di-heme core is colored in each model.
(TIF)

S2 Fig Multiple amino acid sequence alignment of RoxA, PA2077 and PA2078 obtained by T-COFFE. The amino acids shown in the model of Fig 3 are highlighted by green boxes.
(TIF)

S3 Fig MS/MS spectrum (m/z 313→full scan) of the diol 7,10-DiHOME released from 10-H(P)OME by wild type PA2078 and mutant PA2078 H365Q. The same conversion pattern was obtained for all PA2078 mutants (H130Q, H365Q and C518S), indicating that the mutated residues are not involved in activity.
(TIF)

S1 Table Bacterial strains used in this study. (DOCX)

S2 Table Primers used for directed mutagenesis and sequencing. (DOCX)
