# Supplementary figures and images for: In Silico/In Vivo Insights into the Functional and Evolutionary Pathway of Pseudomonas aeruginosa Oleate-Diol Synthase. Discovery of a New Bacterial Di-Heme Cytochrome C Peroxidase Subfamily
Source: PLoS One. 2015 Jul 8;10(7):e0131462. doi: 10.1371/journal.pone.0131462 (PMC4496055; doi:10.1371/journal.pone.0131462)

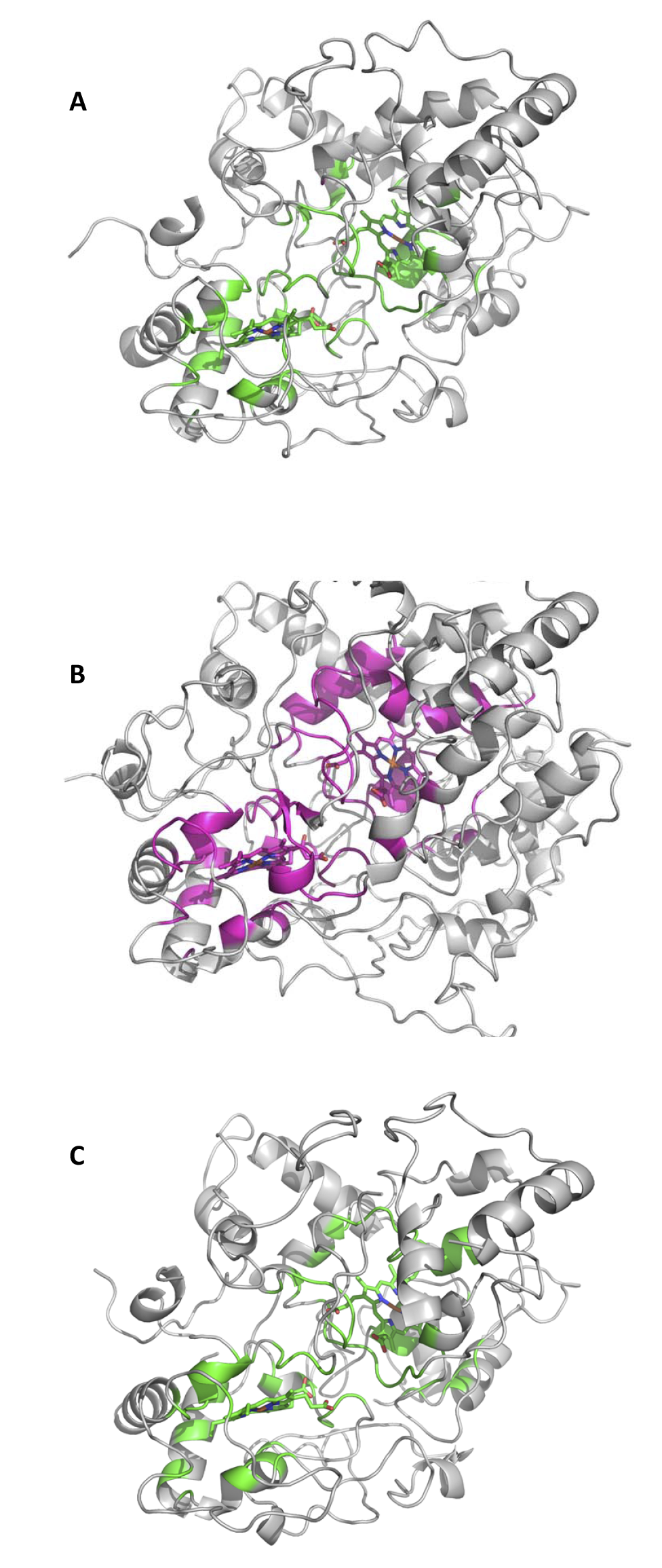

Supplement: S1 Fig — The structure of RoxA (B), used as a template, is shown as a reference. Conserved di-heme core is colored in each model. (TIF) [file pone.0131462.s001.tif]

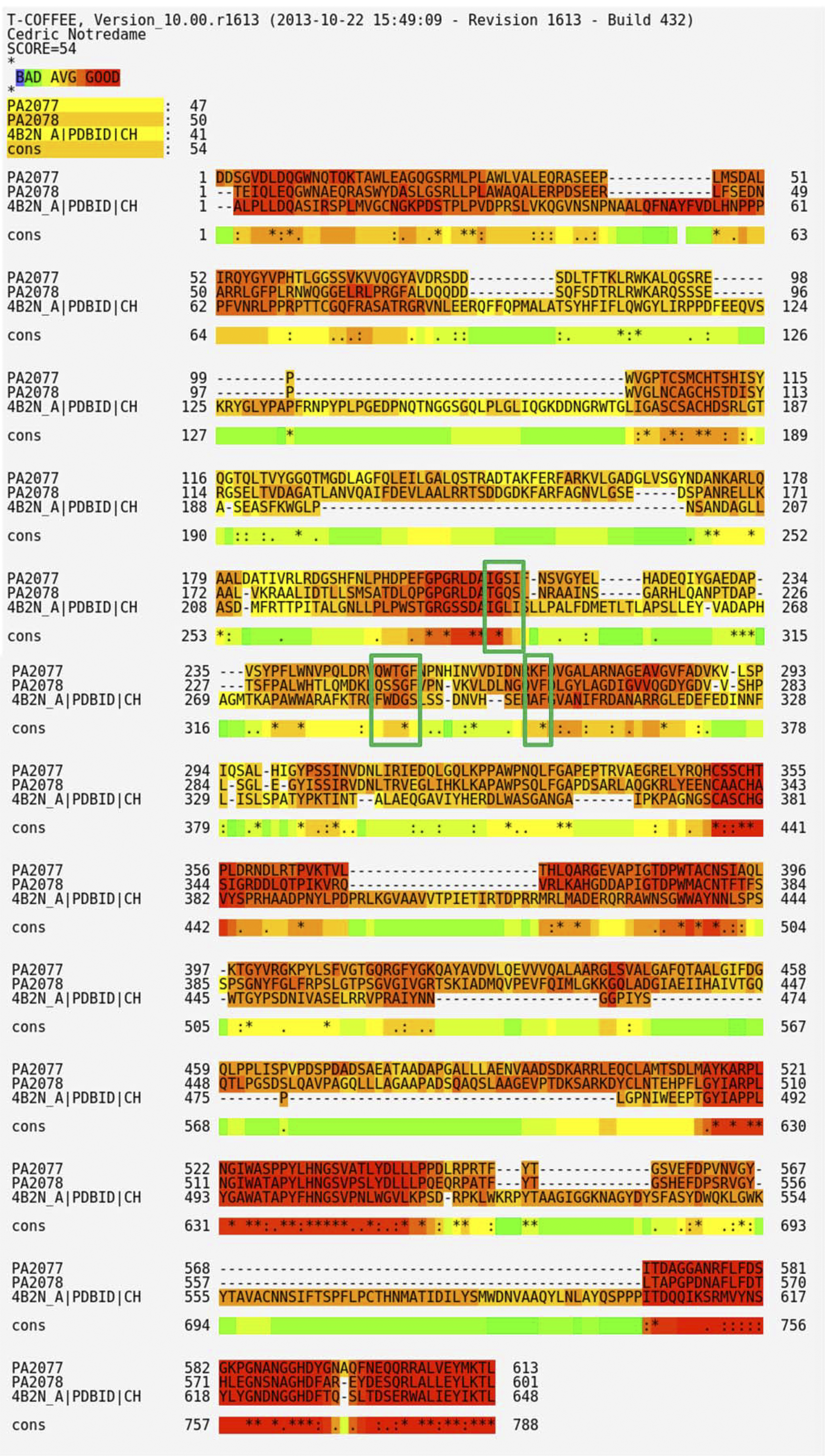

Supplement: S2 Fig — The amino acids shown in the model of Fig 3 are highlighted by green boxes. (TIF) [file pone.0131462.s002.tif]

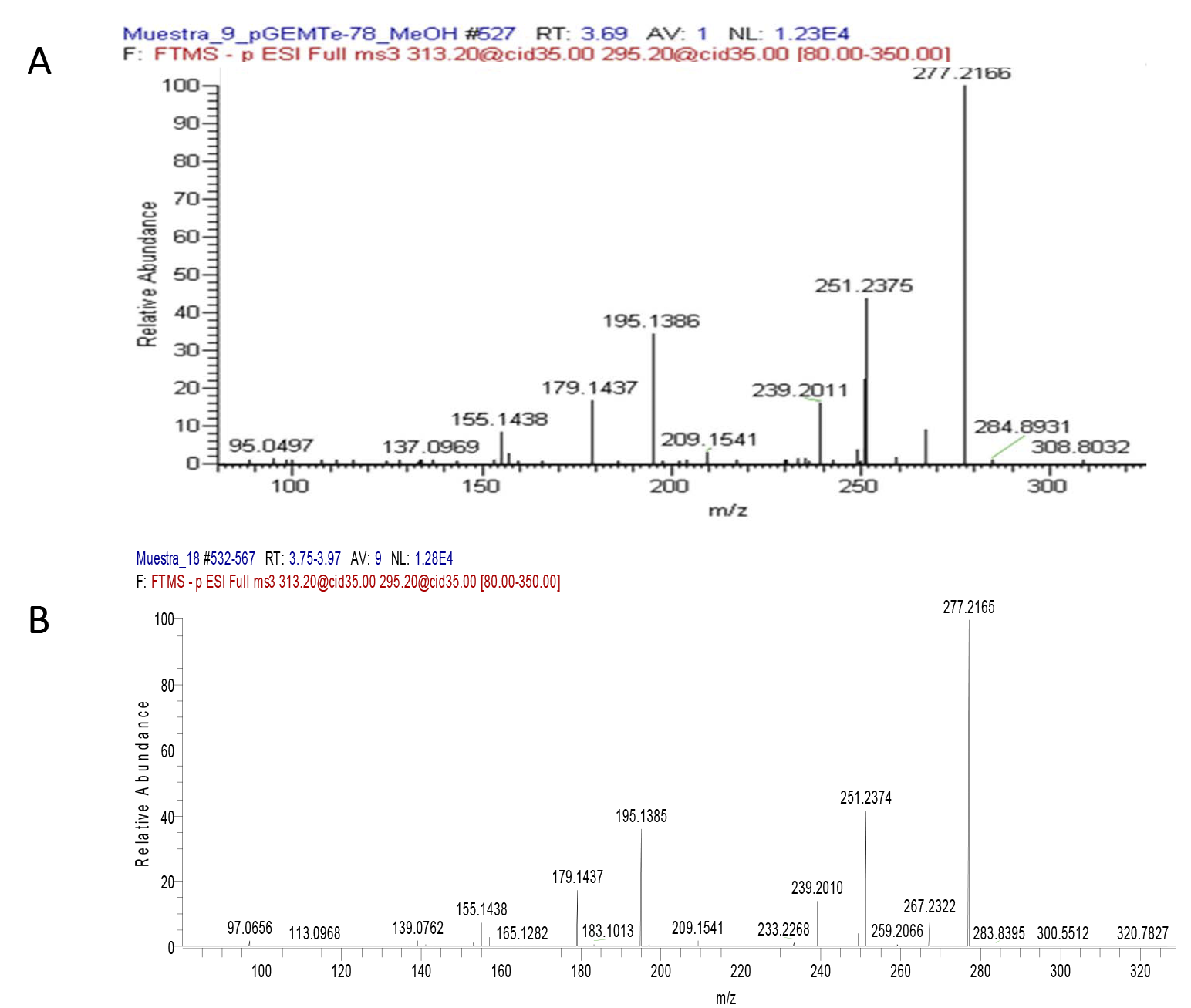

Supplement: S3 Fig — The same conversion pattern was obtained for all PA2078 mutants (H130Q, H365Q and C518S), indicating that the mutated residues are not involved in activity. (TIF) [file pone.0131462.s003.tif]
