# Supplementary material for: In Silico/In Vivo Insights into the Functional and Evolutionary Pathway of Pseudomonas aeruginosa Oleate-Diol Synthase. Discovery of a New Bacterial Di-Heme Cytochrome C Peroxidase Subfamily
Source: PLoS One. 2015 Jul 8;10(7):e0131462. doi: 10.1371/journal.pone.0131462 (PMC4496055; doi:10.1371/journal.pone.0131462)
Supplement: S1 Table — (DOCX) [file pone.0131462.s004.docx]

**Table S1**: **Bacterial strains**

| **Strains** | **Relevant features** | **Reference** |
| --- | --- | --- |
| *P. aeruginosa* PAO1 strains |  |  |
| PAO1 | Wild-type, positive control strain | UWGS |
| ΔPA2077/77 | Tet^r^ Cm^r^ ΔPA2077 mutant carrying pMMB-77 | [3] |
| ΔPA2077/78 | Tet^r^ Cm^r^ ΔPA2077 mutant carrying pMMB-78 | [3] |
| ΔPA2078/77 | Tet^r^ Cm^r^ ΔPA2078 mutant carrying pMMB-77 | [3] |
| ΔPA2078/78 | Tet^r^ Cm^r^ ΔPA2078 mutant carrying pMMB-78 | [3] |
| ΔPA2077/77+78 | Tet^r^ Cm^r^ ΔPA2077 mutant carrying pMMB-77+78 | This work |
| ΔPA2078/77+78 | Tet^r^ Cm^r^ ΔPA2078 mutant carrying pMMB-77+78 | This work |
| *P. aeruginosa* KK strains | Isolated from a cystic fibrosis (CF) patient at different stages of disease |  |
| KK1,KK14, KK72 | Primo-colonization, 18 and 156 months later, respectively | [56] |
