# Supplementary material for: In Silico/In Vivo Insights into the Functional and Evolutionary Pathway of Pseudomonas aeruginosa Oleate-Diol Synthase. Discovery of a New Bacterial Di-Heme Cytochrome C Peroxidase Subfamily
Source: PLoS One. 2015 Jul 8;10(7):e0131462. doi: 10.1371/journal.pone.0131462 (PMC4496055; doi:10.1371/journal.pone.0131462)
Supplement: S2 Table — (DOCX) [file pone.0131462.s005.docx]

**Table S2**: **Primers used in this study**

| **Primers** | **Sequence** |
| --- | --- |
| 77H130QF | GACCTGCAGCATGTGC*CA****G***ACCTCGCACATCAGCTACCAG |
| 77H130QR | CTGGTAGCTGATGTGCGAG*GT****C***TGGCACATGCTGCAGGTC |
| 77H375QF | GGCAACATTGCTCCAGCTGC*CA****G***ACGCCGCTGGACCGCAACG |
| 77H375QR | CGTTGCGGTCCAGCGGC*GT****C***TGGCAGCTGGAGCAATGTTGCC |
| 78H130QF | GGCCGGCTGC*CA****G***TCCACCGACATCAGCG |
| 78H130QR | CGCTGATGTCGGTGGA***C****TG*GCAGCCGGCC |
| 78H365QF | GCGCGGCCTGC*CA****G***GCGAGCATCGGCCGCG |
| 78H365QR | CGCGGCCGATGCTCGC***C****TG*GCAGGCCGCGC |
| 78C518SF | GTGCGCGCAAGGACTA*C****A****GC*CTGAATACCGAGCATCCATTCC |
| 78C518SR | GGAATGGATGCTCGGTATTCAG*GC****T****GT*AGTCCTTGCGCGCAC |

Site-directed mutagenesis modified codons are shown in underlined *italics*. Nucleotide changes are in **bold**.
